# Differences in Transforming Growth Factor‐β1/BMP7 Signaling and Venous Fibrosis Contribute to Female Sex Differences in Arteriovenous Fistulas

**DOI:** 10.1161/JAHA.120.017420

**Published:** 2020-08-06

**Authors:** Chuanqi Cai, Sreenivasulu Kilari, Avishek K. Singh, Chenglei Zhao, Michael L. Simeon, Avanish Misra, Yiqing Li, Sanjay Misra

**Affiliations:** ^1^ Department of Vascular Surgery Union Hospital Tongji Medical College Huazhong University of Science and Technology Wuhan China; ^2^ Vascular and Interventional Radiology Translational Laboratory Department of Radiology Mayo Clinic Rochester MN; ^3^ Department of Vascular Surgery The Second Xiangya Hospital Central South University Changsha Hunan China; ^4^ Department of Biochemistry and Molecular Biology Mayo Clinic Rochester MN; ^5^ Department of Radiology, Vascular and Interventional Radiology Mayo Clinic Rochester MN

**Keywords:** arteriovenous fistula, bone morphogenetic proteins, sexual diversity, transforming growth factor‐β, venous neointimal hyperplasia, Women, Peripheral Vascular Disease, Fibrosis, Stenosis, Vascular Biology

## Abstract

**Background:**

Women have decreased hemodialysis arteriovenous fistula (AVF) maturation and patency rates. We determined the mechanisms responsible for the sex‐specific differences in AVF maturation and stenosis formation by performing whole transcriptome RNA sequencing with differential gene expression and pathway analysis, histopathological changes, and in vitro cell culture experiments from male and female smooth muscle cells.

**Methods and Results:**

Mice with chronic kidney disease and AVF were used. Outflow veins were evaluated for gene expression, histomorphometric analysis, Doppler ultrasound, immunohistologic analysis, and fibrosis. Primary vascular smooth muscle cells were collected from female and male aorta vessels. In female AVFs, RNA sequencing with real‐time polymerase chain reaction analysis demonstrated a significant decrease in the average gene expression of *BMP7* (bone morphogenetic protein 7) and downstream *IL17Rb (interleukin 17 receptor b)*, with increased transforming growth factor‐β1 (*Tgf‐β1)* and transforming growth factor‐β receptor 1 (*Tgfβ‐r1)*. There was decreased peak velocity, negative vascular remodeling with higher venous fibrosis and an increase in synthetic vascular smooth muscle cell phenotype, decrease in proliferation, and increase in apoptosis in female outflow veins at day 28. In vitro primary vascular smooth muscle cell experiments performed under hypoxic conditions demonstrated, in female compared with male cells, that there was increased gene expression of *Tgf‐β1*, *Tgfβ‐r1*, *and* *Col1* with increased migration.

**Conclusions:**

In female AVFs, there is decreased gene expression of *BMP7* and *IL17Rb* with increased *Tgf‐β1* and *Tgfβ‐r1*, and the cellular and vascular differences result in venous fibrosis with negative vascular remodeling.

**Nonstandard Abbreviations and Acronyms**
Arg‐1: arginase 1
AVF: arteriovenous fistula
BMP: bone morphogenetic protein
CD: cluster of differentiation
CKD: chronic kidney disease
GV: graft vein
MMP‐9: matrix metalloproteinase‐9
PCR: polymerase chain reaction
SMA: smooth muscle actin
TGF‐β: transforming growth factor β
TUNEL: terminal deoxynucleotidyl transferase‐mediated dUTP nick‐end labeling

**Clinical Perspective**

**What Is New?**

- In experimental animal models of hemodialysis arteriovenous fistulas, there is increase in gene expression of transforming growth factor‐β with decreased BMP7 (bone morphogenetic protein 7) gene expression in female animals compared with male animals.
- These observations are associated with a decrease in lumen vessel area and increased venous stenosis formation in female animals.

**What Are the Clinical Implications?**

- These observations may explain the differences in venous stenosis formation clinically in women patients.

The arteriovenous fistula (AVF) has been used for several decades for hemodialysis vascular access with 1‐ and 2‐year primary patency rates of 60% and 51%, respectively.^1^ In women, the primary maturation and patency rates are lower and the waiting time before first use of the AVF is longer than in men.^2^, ^3^ It is hypothesized that inflammation, fibrosis, hypoxia, shear stress, cellular proliferation, and migration are responsible for AVF failure.^4^ Anatomic differences in the vein diameter were hypothesized to be the reason for the sex differences. However, Caplin et al measured preoperative vascular diameters and demonstrated that there were no differences in vein size between women and men.^5^

Most experimental and clinical trials have not evaluated the effect of sex on AVF maturation and patency.^6^, ^7^, ^8^, ^9^, ^10^, ^11^, ^12^, ^13^, ^14^ Vascular remodeling and dysfunction after AVF placement is related to extracellular matrix synthesis and degradation, which are key changes that occur with vascular healing.^6^, ^15^, ^16^ Elastin fibers are digested by active matrix metalloproteinase‐9 (MMP‐9),^17^ a process that facilitates adventitial expansion, disrupts basement membranes, and results in endothelial cell, macrophage, vascular smooth muscle cell (VSMC), and fibroblast infiltration, migration, and differentiation.^4^, ^18^, ^19^ The purpose of the present study was to identify the mechanisms and changes in vascular remodeling as assessed using whole transcriptome RNA sequencing with differential gene expression, ultrasound, immunohistologic, and histomorphometric analyses. We compared male outflow veins with female outflow veins using our murine AVF model with chronic kidney disease (CKD). These results have clinical implications in tailoring translational therapies that are sex specific for preventing venous neointimal hyperplasia associated with hemodialysis arteriovenous access (AV).

## Methods

The data that support the findings of this study are available from the corresponding author on reasonable request.

### Experimental Animals

All experiments were conducted with prior approval of Institutional Animal Care and Use Committee of Mayo Clinic (Rochester, MN) and were performed following National Institutes of Health policies and accordance with institutional guidelines. Both sexes of 8‐week‐old C57BL/6J mice were purchased from Jackson Laboratories (Bar Harbor, ME). All mice were housed in a 12/12‐hour light/dark cycle facility with access to food and water ad libitum in Mayo Clinic. A combination of ketamine (120 mg/kg body weight) and xylazine (10 mg/kg body weight) was administered by intraperitoneal injection before all procedures, and anesthesia was maintained by using ketamine (40 mg/kg) and xylazine (3 mg/kg). Before each surgery, one dose of buprenorphine‐sustained release (0.05–0.1 mg/kg body weight) was administered subcutaneously for pain relief.

### CKD and AVF Procedure

CKD with AVF was created as described previously (Figure S1A and S1B).^11^, ^12^, ^13^

### Doppler Ultrasound Examination

The patency of AVFs was evaluated by noninvasive Doppler ultrasound for blood velocity measurement weekly after AVF placement, as described previously.^11^

### Tissue Collection and Processing

Mice were euthanized at different time points (Figure S1C). After overvolume injection of ketamine (120 mg/kg body weight) and xylazine (10 mg/kg body weight), graft vein (GV) and contralateral vein tissue samples were stored in RNA‐later solution (Qiagen, Germany) for gene expression studies or fixed in 4% buffered formalin for histological analysis. Paraffin‐embedded tissue blocks were prepared and cut into 4‐μm thick sections, as described previously.^10^

### VSMC Culture and Hypoxia Stimulation

Female and male 8‐week‐old C57BL/6J mice were anesthetized by overvolume injection of ketamine (120 mg/kg body weight) and xylazine (10 mg/kg body weight). Murine thoracic aortas were collected and VSMCs were isolated by enzymatic digestion and seeded in Dulbecco's modified Eagle's medium (Invitrogen) containing 1% penicillin and streptomycin at 37°C in a humidified 5% CO_2_ atmosphere, as reported elsewhere.^20^ VSMCs from passages 3 to 6 were used. In all experiments, VSMCs were exposed to normoxia and hypoxia stimulation for 6 hours, as reported previously.^10^ Each experiment was repeated 3 times.

### Wound Healing Assay

Primary VSMC migration was also measured by a wound healing assay, according to the protocol previously published.^21^ After 6 hours normoxia or hypoxia stimulation, the scratches were performed with a 100‐μL plastic pipette tip, the cells were washed with PBS, the VSMCs were incubated in free‐serum medium in 12‐well plates, and the wound region was imaged at 0, 12, and 24 hours with a microscope (AMEX‐1100; AMG, Bothell, WA) and analyzed by using ImageJ software.

### Whole Transcriptome RNA Sequence and Differential Gene Expression

Whole transcriptome RNA sequence was performed at the Medical Genome Facility, Mayo Clinic. mRNA libraries were prepared using TruSeq RNA kit V2 (Illumina, Inc, San Diego, CA), and mRNA libraries were loaded to TruSeq v3 paired end flow cells (Illumina, Inc) in such a way that generates 100 million total reads/sample. The flow cells were then sequenced on HiSeq 2000/2500 using TruSeq SBS sequencing kit version 3 (Illumina, Inc), and the data were collected using HiSeq data collection version 2.0.12.0 software. Base calling was performed using Illumina's RTA version 1.17.21.3. Further data analysis was performed using the MAPRSeq v.1.2.1 system, the Bioinformatics Core standard tool, Top Hat 2.0.6, and Feature Counts software. Gene expression was standardized to 1 million reads and normalized for gene length (reads per kilobase pair per million mapped reads). The mean reads per kilobase pair per million mapped reads of each gene of the male were divided with the female to obtained gene expression change in males versus females. The differentially regulated mRNA list was then uploaded to Gene‐E (https://software.broadinstitute.org/GENE‐E) to create a heat map. Gene ontology and network analysis of differentially expressed genes were performed using Web Gestalt (http://www.webgestalt.org/), and the upstream regulator analysis was performed using ingenuity pathway analysis (QIAGEN Inc; https://digitalinsights.qiagen.com/products‐overview/discovery‐insights‐portfolio/analysis‐and‐visualization/qiagen‐ipa/).

### cDNA Synthesis and Real‐Time Polymerase Chain Reaction

RNA was isolated using miRNeasy kit (Qiagen, Germany), and cDNA was synthesized using an iScript kit (Bio‐Rad, CA). Polymerase chain reactions (PCRs) were performed using iTaq universal SYBR Green Master Mix (Bio‐Rad), and cycle quantification (cq) values were measured by Bio‐Rad CFX Manager software (Bio‐Rad). The primers used for PCR are listed in Table S1. The ∆cq values of GVs and contralateral veins were normalized to *TBP1* reference gene, and tubulin was used as reference gene in the VSMC cell culture study. The fold change in gene expression was calculated following the 2^−(ΔΔCT)^ method, as described previously.^13^

### Immunohistochemistry and Immunofluorescence Staining

Immunohistochemistry and immunofluorescence staining were performed on paraffin‐embedded sections from AVF vessels using the EnVision (Dako, CA) method after heat‐induced antigen retrieval. The antibodies and their source were listed in Table S2. The peroxidase activity was visualized using 3,3′‐diaminobenzidine for immunohistochemistry staining. Prolong Gold antifade reagent with 4',6‐diamidino‐2‐phenylindole (Invitrogen, IL) was used for nuclear staining and mounting for immunofluorescence staining. All images were captured using Axio Imager M2 microscope (Carl Zeiss, Germany).

### Masson Trichrome Staining and Terminal Deoxynucleotidyl Transferase‐Mediated dUTP Nick‐End Labeling Assay

Masson trichrome (Richard‐Allan Scientific, Kalamazoo, MI) and terminal deoxynucleotidyl transferase‐mediated dUTP nick‐end labeling (TUNEL) staining (TACS 2 TdT DAB In Situ Apoptosis Detection Kit; Trevigen Inc, Gaithersburg, MD) were performed on female and male outflow veins, according to the manufacturer's directions, to evaluate vascular fibrosis and apoptosis, respectively.^11^, ^12^, ^13^

### Morphometric and Image Analysis

Hematoxylin and eosin stained outflow veins to assess venous remodeling were assessed. Images were digitized to capture a minimum of 1936×1460 pixels, covering one entire cross‐section using an M2 Microscope (Carl Zeiss) with an Axiocam 503 color camera (Carl Zeiss). These slides were analyzed using ZEN 2 blue edition version 2.0 (Carl Zeiss), as described elsewhere.^9^, ^10^, ^14^, ^15^, ^16^ In addition, slides stained for Masson trichrome, TUNEL, smooth muscle actin (SMA), cluster of differentiation (CD) 68, inducible NO synthase (iNOS), myosin heavy chain (MYH11), arginase 1 (Arg‐1), fibroblast specific protein‐1 (FSP‐1), phospho Mothers Against Decapentaplegic Homolog 3 (pSMAD3), MMP‐9, collagen 1, and Ki‐67 were digitized and quantified, as described elsewhere.^9^, ^10^, ^14^, ^15^, ^16^ The index for each stain (TUNEL, SMA, CD68, iNOS, MYH11, Arg‐1, FSP‐1, pSMAD3, MMP‐9, collagen 1, and Ki‐67) was calculated as follows: (number of total cells positive for that stain/total number of cells)×100.

### Assessment of Serum Blood Urea Nitrogen and Creatinine Levels

Serum blood urea nitrogen and creatinine were determined as described elsewhere.^11^, ^12^, ^13^

### Statistical Analysis

All data were expressed as mean±SEM. We used Kaplan‐Meier survival model to assess for differences in patency between male and female animals using a Mantel‐Cox test. ANOVA with repeated measurements with Bonferroni correction or two‐sample *t* test was performed. The mRNAs with a fold change of >1.5 and <0.75 were considered upregulated and downregulated, respectively. The level of significance was set at not significant (*P*>0.05), *P*<0.05, *P*<0.01, *P*<0.001, or *P*<0.0001. GraphPad Prism version 8 (GraphPad Software Inc, CA) was used for all statistical analysis.

## Results

### Surgical Outcomes

**Figure 1:**
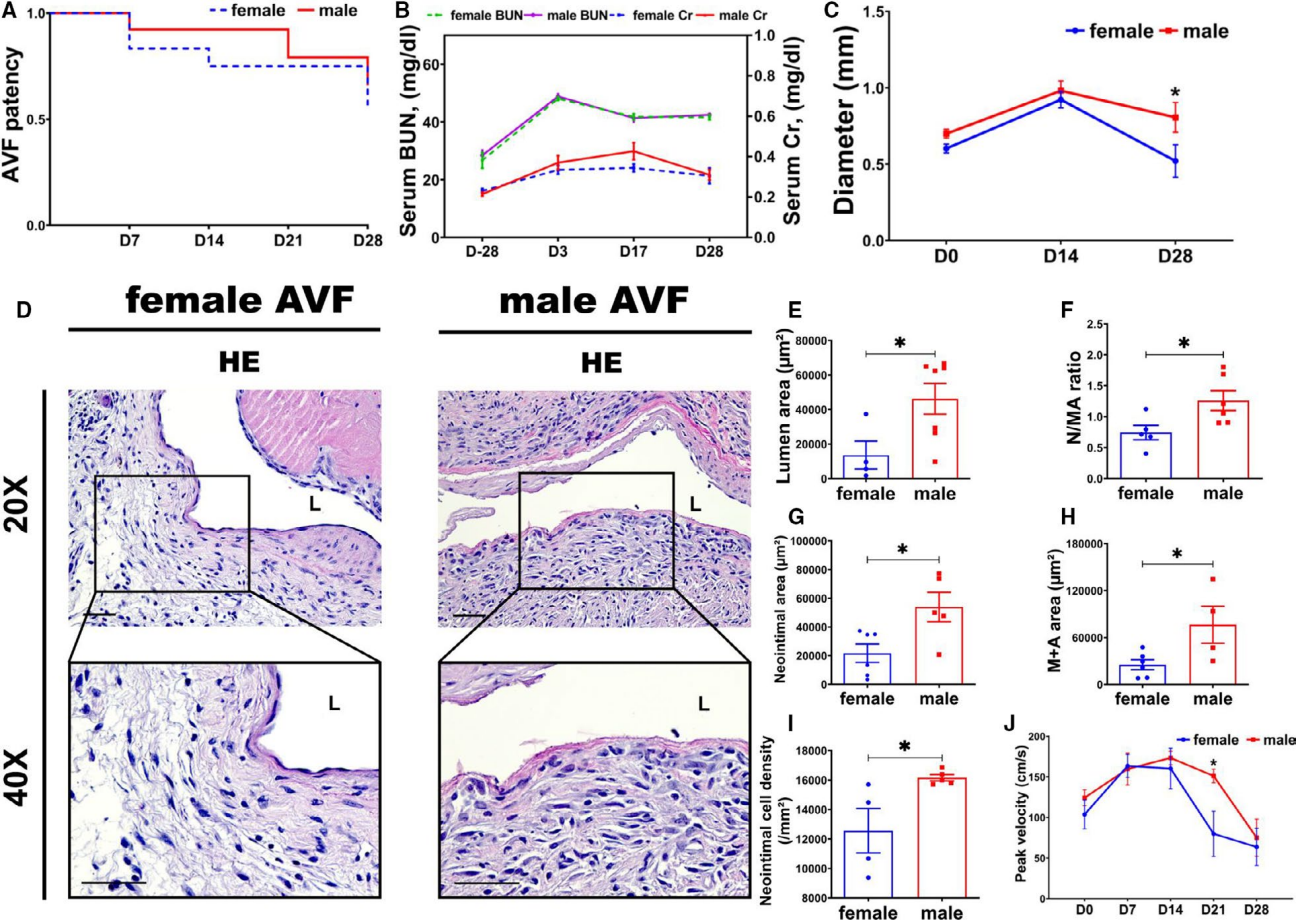
Murine outcomes of arteriovenous fistulas (AVFs). **A**, The overall patency rate of female AVFs (N=18) was lower than males (N=19). **B**, After chronic kidney disease surgery, the average blood urea nitrogen (BUN) and creatinine (Cr) increased significantly in both males (N=7) and females (N=6). There was no difference on the basis of sex. **C**, At day (D) 28 after AVF placement, there was a significant increase in the average outflow vein diameter in male animals (N=7) compared with females (N=6). **D**, Representative hematoxylin and eosin (HE) sections from female and male AVFs at day 28. In female AVFs compared with males, there was significant decrease in the average lumen vessel area (female, N=4; male, N=7) (**E**), neointimal area/(media+adventitial area) (N/MA) ratio (female, N=5; male, N=6) (**F**), neointimal area (female, N=6; male, N=5) (**G**), media area plus adventitia area (M+A) (female, N=6; male, N=4) (**H**), and neointimal cell density (female, N=4; male, N=5) (**I**). **J**, At day 21, the average peak velocity of male AVFs was significantly higher (female, N=4; male, N=6). ANOVA with repeated measures with Bonferroni correction was performed (**B**, **C**, and **J**). Mantel‐Cox test was performed (**A**). Two‐sample *t* test was performed (**E** through **I**). Significant differences between female and male at the respective time points are indicated. *P<0.05. Bar=50 μm.

Thirty‐seven C57BL/6J mice were used in this study, including 18 females and 19 males. AVF patency detected by Doppler ultrasound showed a decrease in females (78%) compared with males (84%) at day 28 (*P*=0.05; Figure 1A).

### Biochemical Measures in Female and Male Mice Following Partial Nephrectomy and AVF Surgery

After partial nephrectomy, there was a significant increase in the average blood urea nitrogen and creatinine in both male and female animals compared with baseline values, with no sexual difference (Figure 1B).

### Significant Inward Venous Remodeling in Female AVFs Compared With Males

The temporal changes in the diameter of outflow veins were determined by intraoperative measurements after AVF creation at 14 and 28 days after creation (Figure 1C). At day 28, the average diameter of the outflow veins was significantly smaller in female animals compared with male animals (female, 0.52±0.11 mm; male, 0.81±0.10 mm; average decrease, 36%; *P*<0.05; Figure 1C). Morphometric analysis on hematoxylin and eosin stained outflow vein sections (Figure 1D) demonstrated that the average lumen vessel area was significantly smaller in female animals compared with male animals (female, 13 589.39±8096.31 μm^2^; male, 46 246.05±8926.13 μm^2^; average decrease, 71%; *P*<0.05; Figure 1E). The average neointima area/media+adventitia area ratio was significantly lower in females compared with males (female, 0.74±0.12; male, 1.26±0.16; average decrease, 41%; *P*<0.05; Figure 1F). The average areas of the neointima and media+adventitia were both significantly lower in female outflow veins compared with males (neointima: female, 21 685.52±6386.94 μm^2^; male, 54 238.48±10 368.50 μm^2^; average decrease, 60%; *P*<0.05; Figure 1G; media+adventitia: female, 25 233.29±6376.95 μm^2^; male, 76 403.68±13 711.75 μm^2^; average decrease, 67%; *P*<0.05; Figure 1H). The average cell density of the neointima was significantly reduced as well (female, 12 562.10±1504.19/mm^2^; male, 16 153.21±212.04/mm^2^; average decrease, 22%; *P*<0.05; Figure 1I).

Ultrasound was used to assess the hemodynamic changes after creation of the AVF. At day 21, the average peak velocity was significantly decreased in females compared with males (female, 79.87±27.80 cm/s; male, 149.28±9.14 cm/s; average decrease, 46%; *P*<0.05; Figure 1J).

### Sex‐Dependent Differential Gene Regulation in Mouse AVF Segments

**Figure 2:**
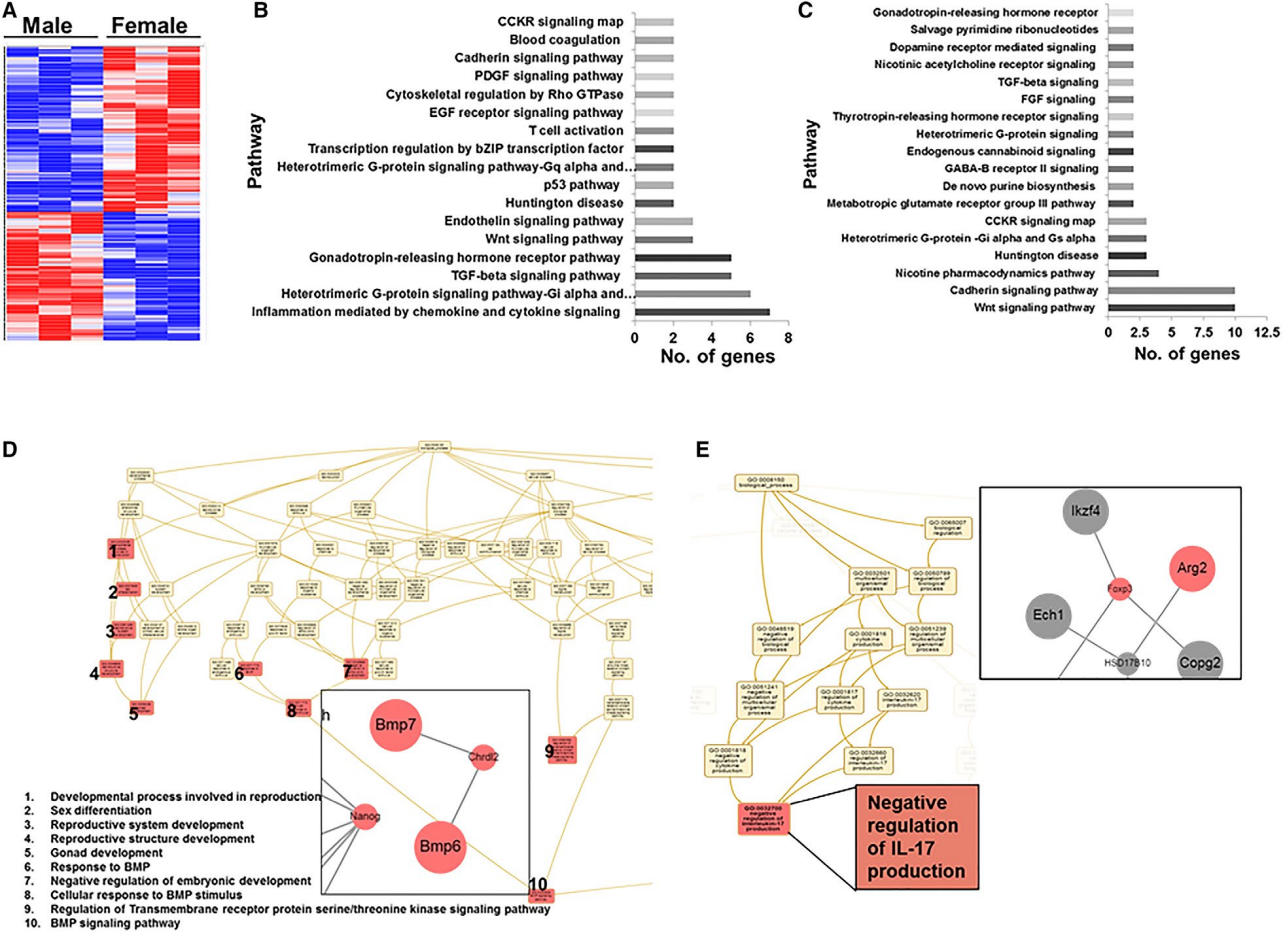
Differential gene expression profile and panther pathway analysis in arteriovenous fistula (AVF) vein segments at day 17. RNA sequencing analysis was performed in outflow veins collected from female and male AVFs at day 17 (female, N=3; male, N=3). **A**, Heat map depicting all common genes that were increased >1.5‐fold (red) or <0.75‐fold (blue) (*P*<0.05). **B** and **C**, Panther pathway analysis of upregulated genes in males (that were downregulated in females) and females (that were downregulated in males), separately. Web Gestalt gene ontology and network analysis of differentially expressed genes upregulated in males (**D**) and in females (**E**). **D** and **E** inset, subnetwork analysis shows BMP7 (bone morphogenetic protein 7) in males, and arginase 2 (Arg2) along with negative regulation of interleukin (IL)‐17 production in females. Upstream regulator analysis was performed using Ingenuity Pathway Analysis tool. CCKR indicates cholecystokinin B receptor; EGF, epidermal growth factor; FGF, fibroblast growth factor; GABA, γ‐aminobutyric acid; PDGF, platelet‐derived growth factor; and TGF‐β, transforming growth factor‐β.

We performed whole transcriptome RNA sequence and differential gene expression using RNA sequencing to assess gene expression changes in male and female outflow veins after CKD and AVF placement. A heat map of the differential gene expression in male versus female at fold change >1.5 with *P*<0.05 is shown (Figure 2A). There were 269 genes that were upregulated >1.5‐fold and 346 genes that were down regulated <0.75‐fold in males compared with females (*P*<0.05). Panther pathway analysis revealed that genes related to inflammation pathway were upregulated in males (Figure 2B). In addition, genes related to wingless and Int‐1 (WNT) and cadherin signaling pathways were upregulated in females (Figure 2C). Web Gestalt pathway analysis tool predicted increased BMP (bone morphogenetic protein) expression in males (Figure 2D) with decreased expression of interleukin 17 being predicted in females (Figure 2E).

### Increased Gene Expressions of *Transforming Growth Factor‐β1* and *Transforming Growth Factor‐β Receptor 1*, But Decreased *Bmp7* and *Interleukin 17 Receptor b,* in Female GVs Compared With Males

**Figure 3:**
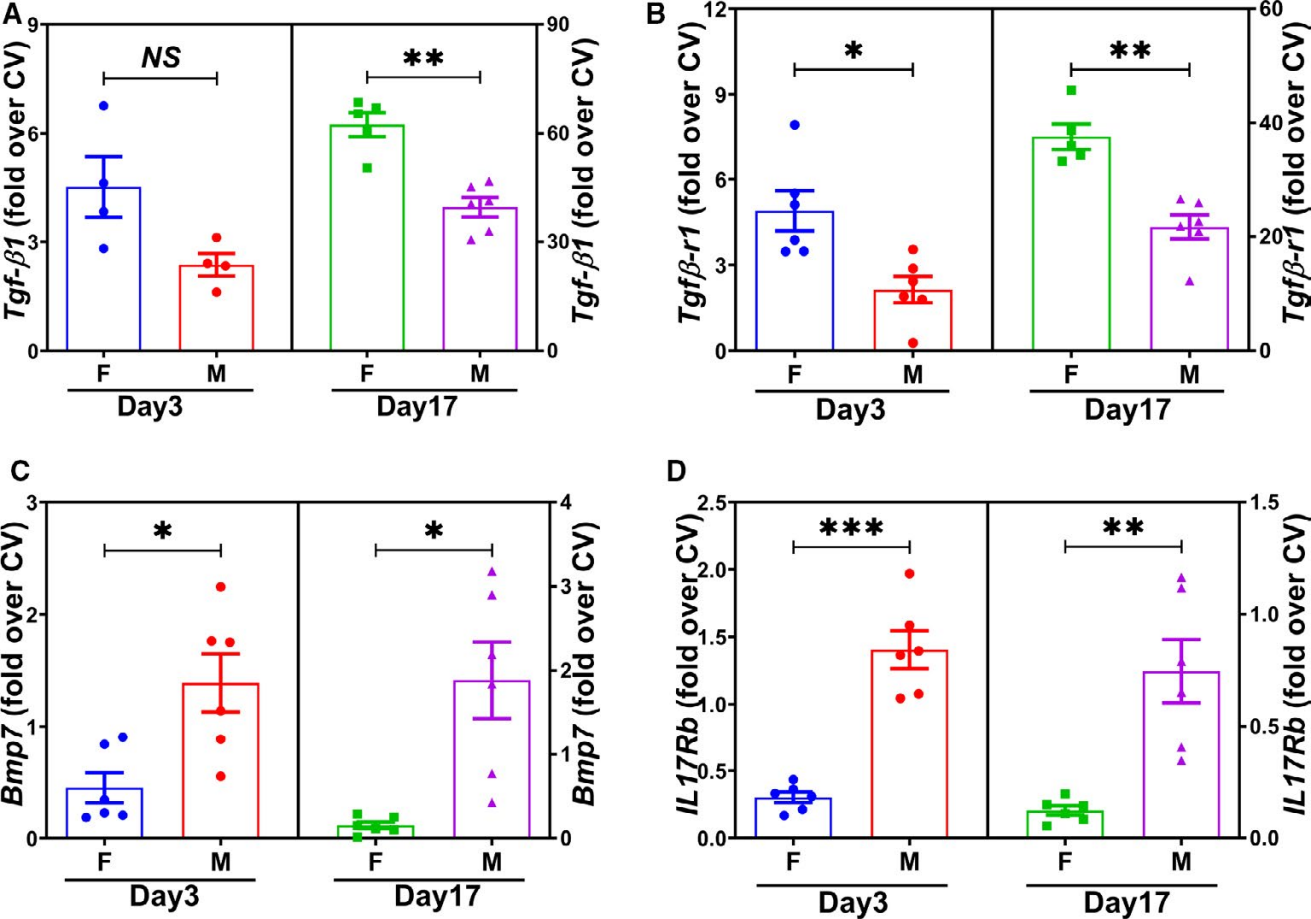
Sexual differences in fibrotic gene expression of arteriovenous fistula vein segments at day 3 and day 17. **A** through **D**, At day 3 and day 17, there was a significant increase in the average gene expression of *Tgfβ1* (day 3: female, N=4; male, N=4; day 17: female, N=5; male, N=6) and *TgfβR1* (day 3: female, N=6; male, N=6; day 17: female, N=5; male, N=6), but decrease in *Bmp7* (day 3: female, N=6; male, N=6; day 17: female, N=6; male, N=6) and *IL17Rb* (day 3: female, N=6; male, N=6; day 17: female, N=6; male, N=6), in female outflow veins compared with males. Four genes were measured at day 3 and 17, and 2‐sample *t* test was performed with Bonferroni correction. Significant differences between female and male at the respective time points are indicated. *P<0.05, **P<0.01, ***P<0.001. CV indicates contralateral vein; F, female; M, male; and NS, not significant.

The gene expression of *Tgf‐β1 (transforming growth factor‐β1)*, *Tgfβ‐r1 (transforming growth factor‐β receptor 1)* *Bmp7 (transforming growth factor‐β receptor 1)*, and *IL17Rb (interleukin 17 receptor b)* was determined using real‐time PCR at day 3 and 17 after AVF creation. At day 3 and day 17, the average gene expression of *Tgf‐β1* (day 3 female *Tgf‐β1*, 4.51±0.84; day3 male *Tgf‐β1*, 2.37±0.31; average increase, 90%; *P*=0.21; Figure 3A; day 17 female *Tgf‐β1*, 62.42±3.29; day 17 male *Tgf‐β1*, 39.54±2.65; average increase, 58%; *P*<0.01; Figure 3A) and *Tgfβ‐r1* (day 3 female *Tgfβ‐r1*, 4.91±0.70; day 3 male *Tgfβ‐r1*, 2.14±0.46; average increase, 129%; *P*<0.05; Figure 3B; day 17 female *Tgfβ‐r1*, 37.60±2.24; day 17 male *Tgfβ‐r1*, 21.70±2.10; average increase, 73%; *P*<0.01; Figure 3B) was significantly increased in female GVs compared with males, whereas gene expression of *Bmp7* (Figure 3C) and *IL17Rb* (Figure 3D) was significantly decreased in female GVs. The average gene expressions of *Bmp7* (day 3 female *Bmp7*, 0.45±0.14; day 3 male *Bmp* *7*, 1.39±0.26; average decrease, 68%; *P*<0.05; Figure 3C; day 17 female *Bmp* *7*, 0.16±0.04; day 17 male *Bmp* *7*, 1.89±0.41; average decrease, 92%; *P*<0.05; Figure 3C) and *IL17Rb* (day 3 female *IL17Rb*, 0.30±0.04; day 3 male *IL17Rb*, 1.41±0.14; average decrease, 79%; *P*<0.001; Figure 3D; day 17 female *IL17Rb*, 0.12±0.02; day 17 male *IL17Rb*, 0.75±0.13; average decrease, 84%; *P*<0.01; Figure 3D) were significantly lower in female outflow veins compared with males.

### Vascular Remodeling and Fibrosis in Outflow Veins on the Basis of Sex

**Figure 4:**
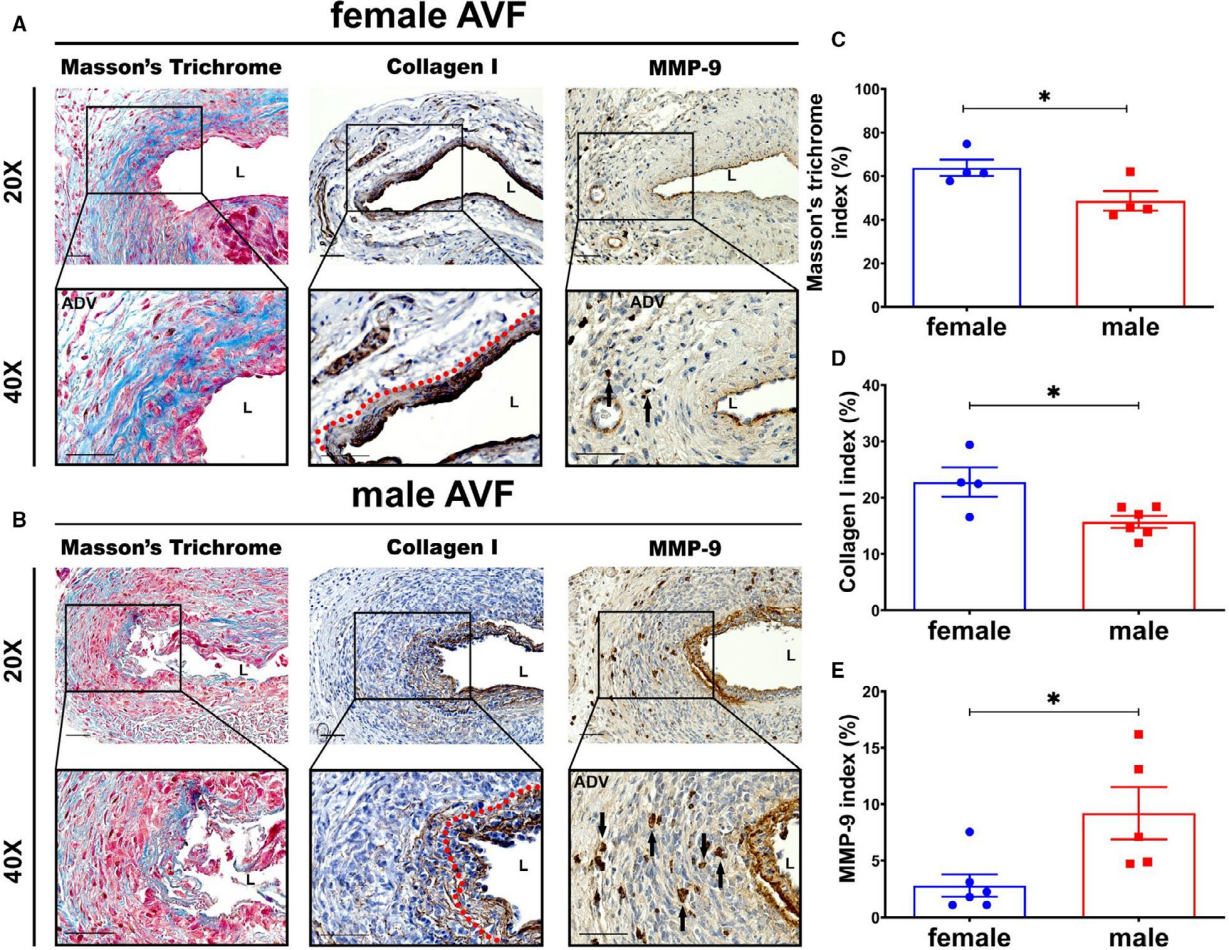
There was increased fibrosis staining in female arteriovenous fistulas (AVFs) compared with males. **A** and **B**, Representative slides for Masson trichrome (female, N=4; male, N=4), collagen I (female, N=4; male, N=6), and matrix metalloproteinase‐9 (MMP‐9) (female, N=6; male, N=5) staining. There were more collagen depositions in female vessels compared with males, but less positive MMP‐9 staining in the female AVFs. **C** through **E**, Semiquantitative analysis showed significant increases in the average Masson trichrome (*P*<0.05) and collagen I (*P*<0.05) indexes, but decrease in MMP‐9 (*P*<0.05) in female outflow veins compared with males. Two‐sample *t* test was performed. Significant differences are indicated. *P<0.05. Positive collagen I and MMP‐9 staining are brown staining. Blue areas indicate positive collagen staining in Masson trichrome staining. Bar=50 μm. Dashed red line indicate positive stained area; and solid arrows, positive cells.

We performed Masson trichrome and collagen I staining to assess differences in fibrosis between females and males (Figure 4A and 4B). Interestingly, both Masson and collagen I staining demonstrated increased staining in the media of the AVF in females compared with males. The average Masson trichrome index was significantly increased in females compared with males (female, 63.83±3.74%; male, 48.69±4.52%; average increase, 132%; *P*<0.05; Figure 4C) as well as the average collagen I index (female, 22.82±2.57%; male, 15.70±1.07%; average increase, 145%; *P*<0.05; Figure 4D). MMP‐9 has been shown to be involved with matrix turnover. The average MMP‐9 index was significantly decreased in females compared with males (female, 2.83±1.00%; male, 9.19±2.32%; average decrease, 69%; *P*<0.05; Figure 4E). In aggregate, these data suggest that there is increased constrictive remodeling involving the media mediated by overexpression of collagen‐1 and decreased turnover with reduced MMP‐9 in female outflow veins compared with males.

### Female AVFs Have Increased Fibrosis, α‐SMA, FSP‐1, and pSMAD3, But Decreased MYH11, Staining

**Figure 5:**
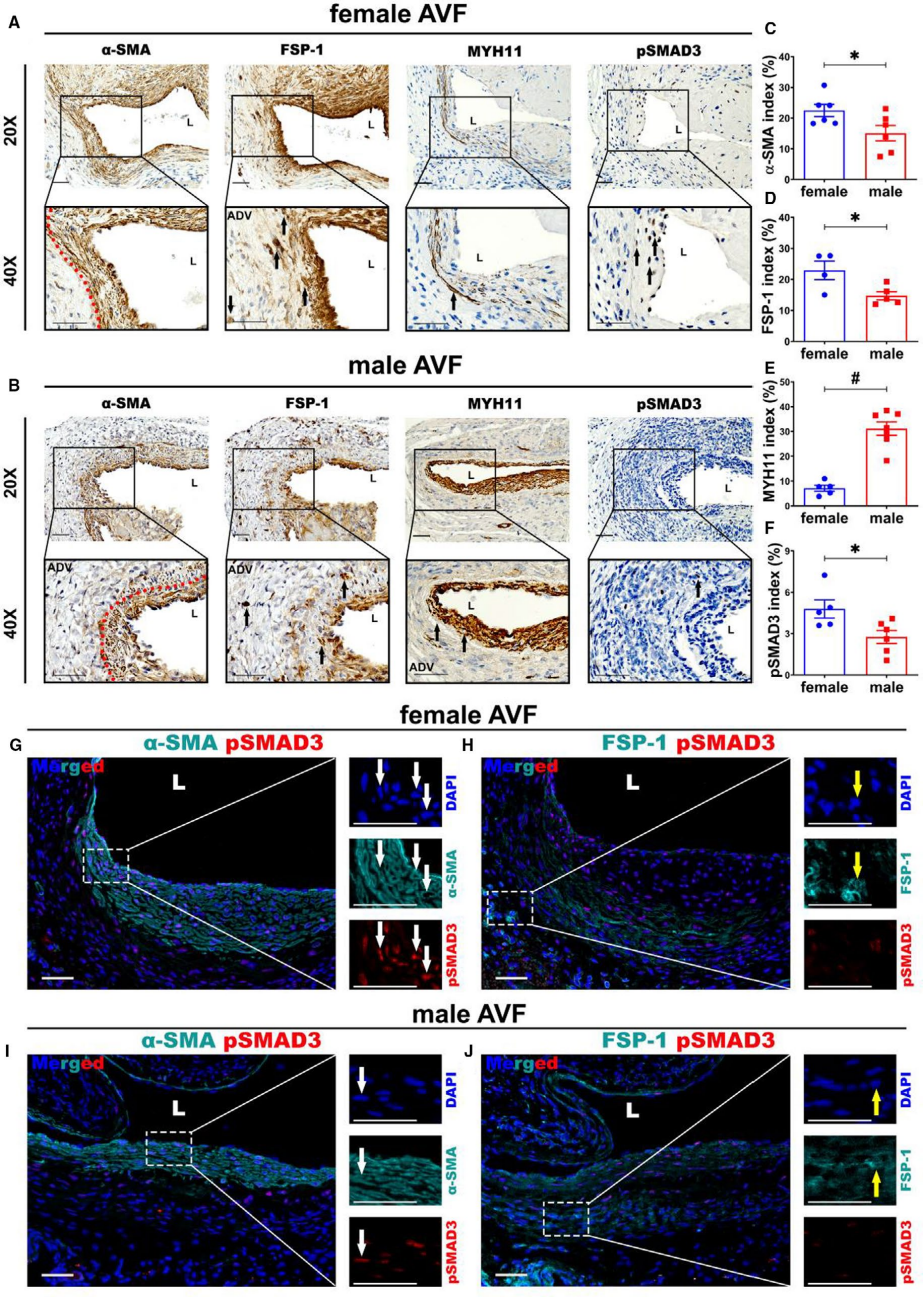
Sexual differences linked to α‐smooth muscle actin (α‐SMA), FSP‐1, MYH11, and pSMAD3 staining. **A** and **B**, Representative slides for α‐SMA (female, N=6; male, N=6), FSP‐1 (female, N=4; male, N=5), MYH11 (female, N=5; male, N=7), and pSMAD3 (female, N=5; male, N=6) staining. Most positive α‐SMA, FSP‐1, MYH11, and pSMAD3 cells were located in the neointimal and media areas in both female and male arteriovenous fistulas (AVFs). **C** through **F**, Semiquantitative analysis showed significant increases in the average α‐SMA (*P*<0.05), FSP‐1 (*P*<0.05), and pSMAD3 (*P*<0.05) indexes, but decrease in MYH11 index (*P*<0.0001), in female outflow veins compared with males. **G** through **J**, Costaining showed more α‐SMA and FSP‐1 positive cells in female vessels. The positive pSMAD3 cells were mainly colocalized with α‐SMA (+) cells, not with FSP‐1 (+) cells. There were more α‐SMA (+)‐pSMAD3 (+) positive cells in female vessels compared with males. Two‐sample *t* test was performed. Significant differences are indicated. *P<0.05, ^#^ *P*<0.0001. Positive α‐SMA, FSP‐1, MYH11, and pSMAD3 immunohistochemistry staining are brown staining. Turquoise indicates positive α‐SMA and FSP‐1 staining. Red indicates pSMAD3 positive staining. Blue indicates positive staining for nuclei. Bar=50 μm. Dashed red line indicates positive stained area; black solid arrows, positive cells; white solid arrows, positive costaining; and yellow solid arrows, negative costaining. ADV indicates adventitia; DAPI, 4',6‐diamidino‐2‐phenylindole; and L, lumen.

Venous neointimal hyperplasia is characterized by increased expression of cells staining positive for α‐SMA and FSP‐1. We determined if there was a difference in the expression of these cells on the basis of sex. Twenty‐eight days after AVF creation, both females and males demonstrated abundant α‐SMA (Figure 5A and 5B) and FSP‐1 staining (Figure 5A and 5B) in the neointima. Semiquantitative analysis demonstrated that there was a significant increase in the average α‐SMA index in females compared with males (female, 22.50±1.98%; male, 15.10±2.53%; average increase, 149%; *P*<0.05; Figure 5C) and average FSP‐1 index (female, 22.90±3.00%; male, 14.73±1.33%; average increase, 155%; *P*<0.05; Figure 5D). We performed MYH11 staining (Figure 5A and 5B), which is the typical marker for contractile phenotype of smooth muscle cells. Semiquantitative analysis demonstrated that there was a significant decrease in average MYH11 index in females compared with males (female, 7.13±1.23%; male, 31.12±2.69%; average decrease, 77%; *P*<0.0001; Figure 5E).

Transforming growth factor (TGF)‐β1/pSMAD3 pathway has been shown to be involved in vascular fibrosis.^22^ We performed pSMAD3 staining, which is the key regulator of TGF‐β pathway. There was a significant increase in the average pSMAD3 index in females (female, 4.79±0.66%; male, 2.98±0.46%; average increase, 61%; *P*<0.05; Figure 5F). To identify the cells that are expressing pSMAD3, we performed costaining of α‐SMA/pSMAD3 and FSP‐1/pSMAD3 (Figures 5G through 5J). Interestingly, pSMAD3 (+) cells were presented throughout the whole vessel wall, with most of the pSMAD3 (+) cells located in the media and neointima. There were more α‐SMA/pSMAD3 costained cells observed in female and male GVs compared with FSP‐1/pSMAD3 costained cells. In aggregate, these data suggest that there was increased constrictive remodeling in female AVFs with venous fibrosis mediated by overexpression of pSMAD3 in female VSMCs.

### Female AVFs Demonstrate Significantly Decreased CD68, Arg‐1, and iNOS Staining Compared With Males

**Figure 6:**
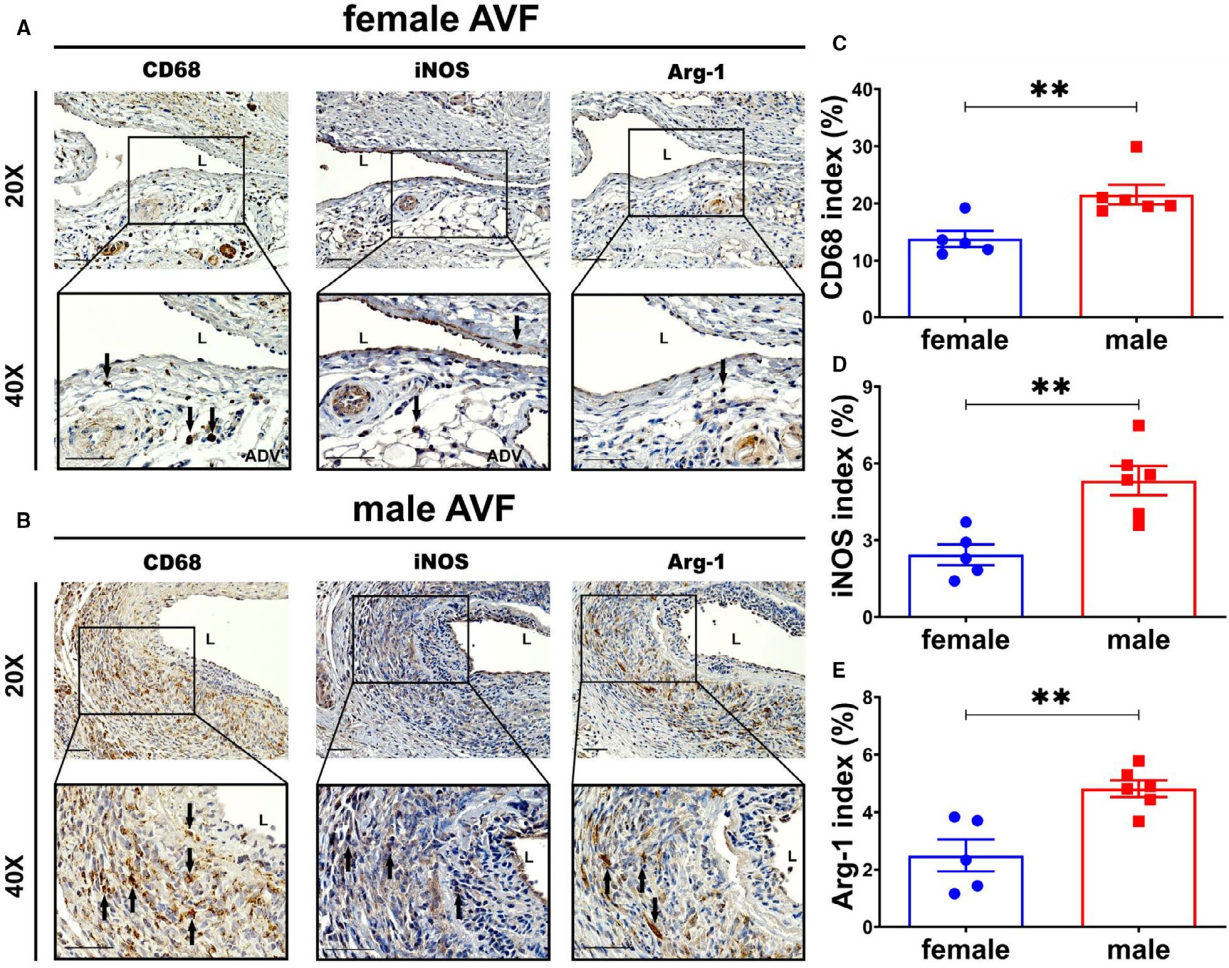
Immunohistochemical staining for inflammation in female and male arteriovenous fistulas (AVFs) at day 28. **A** and **B**, Representative positive cluster of differentiation (CD) 68 (female, N=5; male, N=6), inducible NO synthase (iNOS) (female, N=5; male, N=6), and arginase 1 (Arg‐1) (female, N=5; male, N=6) staining in female and male outflow veins. **C** through **E**, Semiquantitative analysis showed significant decreases in CD68 (*P*<0.01), iNOS (*P*<0.01), and Arg‐1 (*P*<0.01) positive cellular indexes in female outflow veins relative to males. Two‐sample *t* test was performed. Significant differences are indicated. **P<0.01. Positive CD68, iNOS, and Arg‐1 staining is brown staining. Bar=50 μm. Black solid arrows indicate positive cells. ADV indicates adventitia; and L, lumen.

Inflammatory cells, including macrophages (CD68) and M1 (iNOS) and M2 (Arg‐1), are present in venous neointimal hyperplasia associated with AVF failure. Therefore, we determined the expression of these cells using immunostaining. First, we determined if there was a difference in CD68 (+) cells on the basis of sex at the venous neointimal hyperplasia (Figure 6A and 6B). Semiquantitative analysis demonstrated there was a significant reduction in the average CD68 index in females compared with males (female, 13.80±1.42%; male, 21.56±1.71%; average decrease, 36%; *P*<0.01; Figure 6C). Next, iNOS (M1) (Figure 6A and 6B) and Arg‐1 (M2) (Figure 6A and 6B) staining were performed after AVF creation. Semiquantitative analysis demonstrated that in females compared with males, there was a significant decrease in the average iNOS index (female, 2.43±0.41%; male, 5.33±0.57%; average decrease, 54%; *P*<0.01; Figure 6D) and average Arg‐1 index (female, 2.49±0.51%; male, 4.82±0.27%; average decrease, 48%; *P*<0.01; Figure 6E). Moreover, there was significant increase in the average M1/M2 ratio in female AVFs compared with males (female, 2.29±0.51; male, 0.90±0.21; average increase, 254%; *P*<0.05; data not shown). These data demonstrate that there is an increase in proinflammatory macrophages with decrease in CD68 (+) cells in females compared with males.

### Female Outflow Veins Demonstrate Significantly Decreased Proliferation and Increased Apoptosis

**Figure 7:**
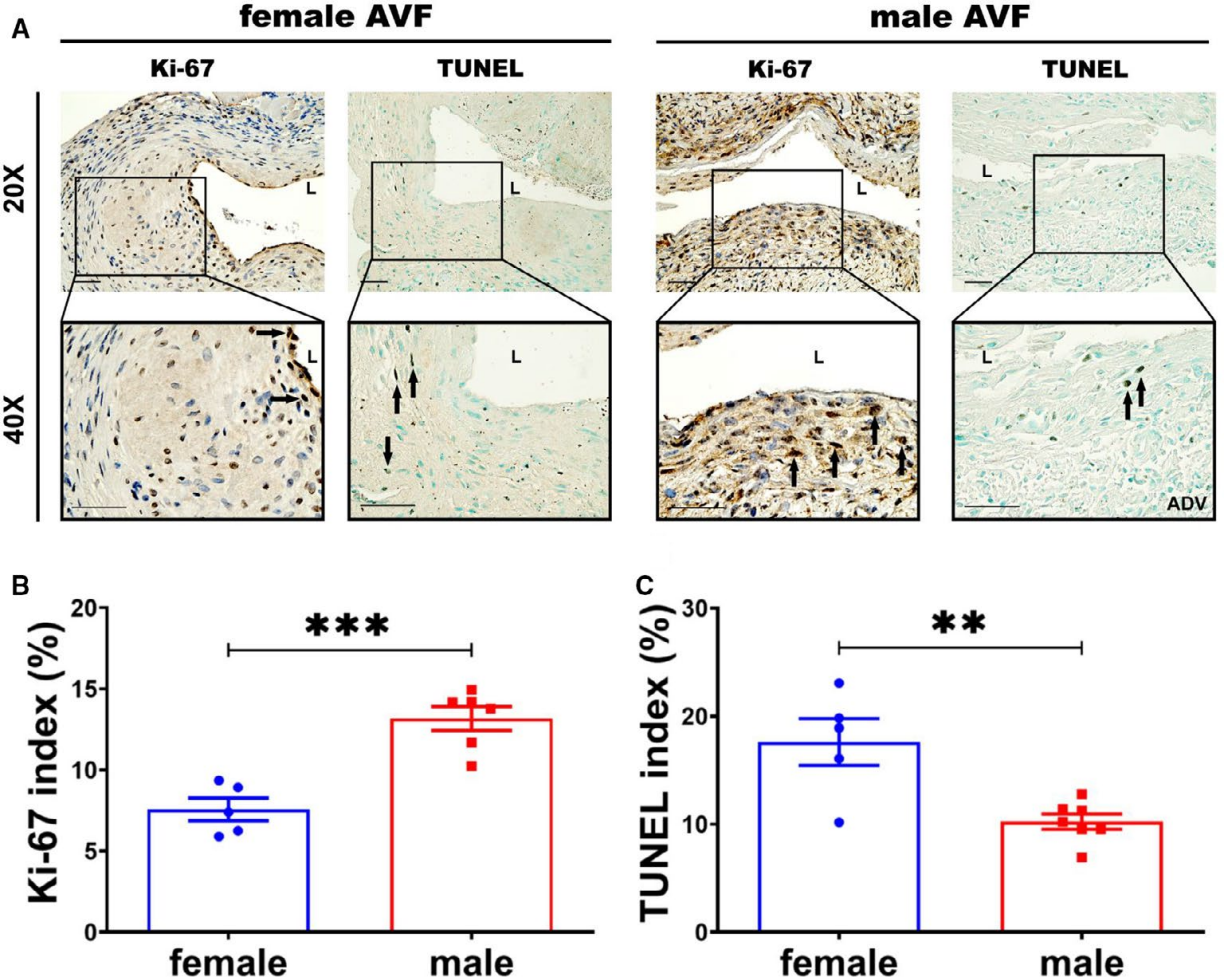
There was decreased proliferation but increased apoptosis in female arteriovenous fistulas (AVFs). **A**, Representative Ki‐67 (female, N=5; male, N=6) and terminal deoxynucleotidyl transferase‐mediated dUTP nick‐end labeling (TUNEL) (female, N=5; male, N=7) sections from female and male AVFs at day 28. Positive Ki‐67 staining was observed throughout the whole male outflow vein. The positive TUNEL staining was shown in the whole vessel wall of female outflow vein. **B** and **C**, There was a significant decrease in the average Ki‐67 staining but increased TUNEL staining in females compared with males at day 28. Two‐sample *t* test was performed. Significant differences between female and male are indicated. **P<0.01, and ***P<0.001. Positive Ki‐67 and TUNEL staining is brown staining. Bar=50 μm. Black solid arrows indicate positive cells. ADV indicates adventitia; and L, lumen.

We wanted to determine if proliferation and apoptosis were different by using Ki‐67 staining and TUNEL staining (Figure 7). We assessed proliferation using Ki‐67 staining (Figure 7A) and apoptosis using TUNEL staining (Figure 7A). There was a significant decrease in the average Ki‐67 index of female outflow veins compared with males (female, 7.04±0.63%; male, 13.18±0.74%; average decrease, 47%; *P*<0.001; Figure 7B). There was a significant increase in the average TUNEL index of females compared with males (female, 17.62±2.17%; male, 10.24±0.71%; average increase, 172%; *P*<0.01; Figure 7C).

### Diverse Characterization of Female and Male VSMCs

**Figure 8:**
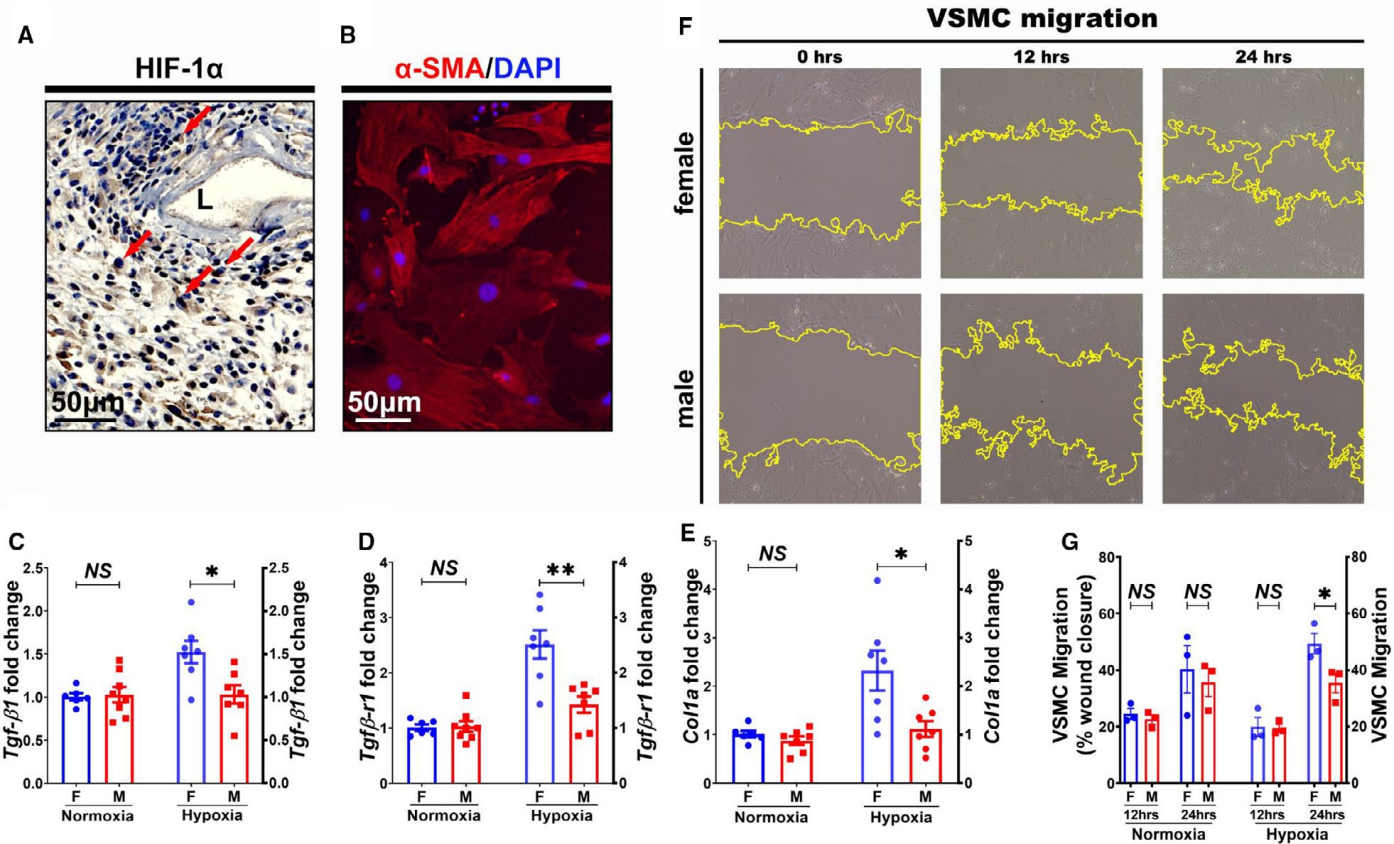
Vascular smooth muscle cell (VSMC) differences for female and male mice. **A**, Representative slides for hypoxia‐inducible factor‐1α (HIF‐1α) staining of outflow vein at day 14 after arteriovenous fistula creation. **B**, VSMCs were stained as positive α‐smooth muscle actin (α‐SMA). **C** through **E**, Three independent experiments presented significant increase in the average gene expression of *Tgfβ1*, *TgfβR1*, and *Col1* in female hypoxia induced VSMCs compared with males, but no significant differences in normoxia condition. **F**, VSMC migration was assayed at 12 and 24 hours after 6 hours of hypoxia stimulation. **G**, In normoxia condition, there was no significance of VSMC migration at 12 and 24 hours. In hypoxia condition, there was significantly faster VSMC migration in female cells at 24 hours compared with males, but no significant difference at 12 hours. Three genes were measured, and 2‐sample *t* test with Bonferroni correction was performed (**C** through **E**). ANOVA with repeated measurments was performed (**G**). Significant differences are indicated. *P<0.05, **P<0.01. Positive HIF‐1α immunohistochemistry staining is brown staining. Red indicates α‐SMA positive staining. Blue indicates positive staining for nuclei. Bar=50 μm. Dashed red line indicates positive stained area; and solid arrows, positive cells. DAPI indicates 4',6‐diamidino‐2‐phenylindole; F, female; L, lumen; M, male; and NS, not significant.

Our previous results demonstrated that hypoxia is a key regulator of AVF malfunction pathology.^9^ In our AVF model, positive hypoxia‐inducible factor‐1α staining was confirmed at day 14 (Figure 8A). In addition, there was no significant difference of hypoxia‐inducible factor‐1α staining between female and male AVFs at day 28 (Figure S2C). We wanted to determine whether our in vivo sexual difference was linked to difference in TGF‐β1 signaling in VSMCs. Primary cultured VSMCs were confirmed by positive α‐SMA staining (Figure 8B). The gene expression of *Tgf‐β1*, *Tgfβ‐r1*, and *Col1a* was determined using real‐time PCR at 6 hours after hypoxia/normoxia stimulation in female and male passage 3 VSMCs. We determined the gene expression of *Tgf‐β1*, *Tgfβ‐r1*, and *Col1a* in normoxic conditions using real‐time PCR. There was no significant difference in the average gene expression of *Tgf‐β1* (female VSMC, 1.00±0.04; male VSMC, 1.03±0.09; *P>*0.05; Figure 8C), *Tgfβ‐r1* (female VSMC, 1.01±0.06; male VSMC, 1.03±0.10; *P>*0.05; Figure 8D), and *Col1a* (female VSMC, 1.01±0.07; male VSMC, 0.88±0.09; *P>*0.05; Figure 8E) of female normoxia VSMCs compared with male normoxia cells. Next, we determined the gene expression of *Tgf‐β1*, *Tgfβ‐r1*, and *Col1a* in hypoxic condition in female VSMCs compared with male cells (Figure 8C through 8E). There was significant increase in the average gene expression of *Tgf‐β1* (female VSMC, 1.52±0.13; male VSMC, 1.03±0.11; average increase, 48%; *P*<0.05; Figure 8C), *Tgfβ‐r1* (female VSMC, 2.52±0.26; male VSMC, 1.42±0.15; average increase, 77%; *P*<0.01; Figure 8D), and *Col1a* (female VSMC, 2.32±0.41; male VSMC, 1.12±0.17; average increase, 107%; *P*<0.05; Figure 8E) in female hypoxia stimulated VSMCs compared with male hypoxia stimulated cells.

In addition, increased TGF‐β is linked to promotion in VSMC migration.^23^ We determined the VSMC migration after 6 hours of hypoxia stimulation (Figure 8F). There was no significant difference in the VSMC migration under normoxic condition at 12 and 24 hours (Figure 8G). In the hypoxia group, there was no sexual difference in the VSMC migration at 12 hours. At 24 hours, female hypoxia stimulated VSMCs showed faster migration rate compared with male cells (female wound closure, 49.32±3.64%; male wound closure, 35.47±3.50%; average increase, 39%; *P*<0.05; Figure 8G). On the basis of this finding, we postulate that in vivo AVF sex differences were at least partially linked to in vitro cellular differences for TGF‐β1 signaling.

## Discussion

In the present study, we determined the differences in vascular remodeling after the placement of an AVF in male and female mice with CKD. We performed RNA sequencing that identified that female veins are more fibrotic than males because of a decrease in BMP7 gene expression with increase in TGF‐β1 and TGF‐βR1, and decrease in interleukin 17 receptor b (IL17Rb). Histologically, female animals have negative vascular remodeling with increased medial fibrosis, increased staining of α‐SMA and FSP‐1, and decreased staining for inflammatory cellular markers. Female outflow veins have increased cell apoptosis and decreased proliferation.

Extensive research has been done on AVF failure in animal models, but female animals have not been studied.^6^, ^7^, ^8^, ^10^, ^24^ Clinical observation has demonstrated that women patients with AVFs are more susceptible to failure than men.^2^ Experimental data demonstrate that estrogen enhances the elastin/collagen ratio in rat aorta.^25^ Studies on vascular dilatation demonstrate that less enlargement occurs in female rats. This process has been attributed to estrogen‐mediated decreases in inflammation and MMP‐9 generation.^26^ One clinical study demonstrates that women undergoing hormone replacement treatment have fewer CD68 (+) cells, lower levels of MMP‐9, and more collagen deposition in carotid arterial plaques compared with women not receiving such treatment.^27^ In the present study, disparities between female and male murine AVFs were illustrated in a murine model. At day 0, there was no significant difference in average outflow venous diameter after AVF creation in females and males. Sustained Doppler monitoring and evaluation proved that earlier hemodynamic change with a decrease in peak velocity occurred in female mice at day 21 after AVF placing. Female AVFs demonstrated more α‐SMA (+) and FSP‐1 (+) cells with increased medial fibrosis, with decreased MMP‐9 expression. There were fewer inflammatory cells (both M1 and M2 cells), less cell proliferation (Ki‐67), and increased apoptosis.

Whole transcriptome analyses revealed that the genes involved in inflammation and BMP6/7 pathway were downregulated in females compared with males. Genes targeted by BMP6/7 pathway are known as suppressors for tissue fibrosis. BMP7 is a known antagonist of downstream TGF‐β signaling mediated through an SMAD dependent pathway.^28^ In addition, MMP‐9 was found to be decreased, which is involved in matrix degradation and thus has antifibrotic function.^29^ There was an increase in α‐SMA and FSP‐1 in females compared with males, with the decrease in antifibrotic genes suggesting that females could have more tissue fibrosis compared with males.

Network analysis predicted a decrease in interleukin‐17 in females with an increase of IL17Rb in males, which was confirmed using real‐time PCR analysis. Endothelial‐to‐mesenchymal transition is known to contribute to stenosis formation after AVF creation.^30^ Our previous data prove that TGF‐β is able to accelerate endothelial‐to‐mesenchymal transition process in female AVF after angioplasty procedure.^31^ This is consistent with our findings in female AVFs at day 28, where there was increased α‐SMA (+) and FSP‐1 (+) staining with less MYH11 (+) staining. Excessive TGF‐β1 expression strongly inhibits cellular proliferation and has proapoptotic properties.^32^ This is consistent with the decreased Ki‐67 staining but increased TUNEL staining in females in vivo. Interestingly, BMP7 has been shown to be involved in inhibiting endothelial‐to‐mesenchymal transition process in vitro and in vivo.^33^, ^34^ This is consistent with our findings in male AVFs at day 28 that there was decreased α‐SMA and FSP‐1 (+) staining. This indicates that the sex difference is linked to increase in endothelial‐to‐mesenchymal transition process in females and partially modulated by TGF‐β1 and BMP7 pathway.

Differences in functional outcomes of VSMCs isolated from male and female animals have been studied in pulmonary hypertension and atherosclerosis diseases.^35^, ^36^ These studies have demonstrated that there is differential function of these cells on the basis of the sex of the animal. In the present study, we examined the role of TGF‐β1 in hypoxic and normoxic VSMCs isolated from male and female animals. Previous study from our laboratory showed that simvastatin and vascular endothelial growth factor‐A reduction could reduce fibroblast differentiation into smooth muscle cells under hypoxic stress.^10^, ^12^, ^37^ We observed that under hypoxic conditions, there was a significant increase in the gene expression of TGF‐β1, TGF‐βR1, and Col1a in female VSMCs compared with male. Previous articles have described that hypoxia can upregulate TGF‐β1 and Col1a.^38^ We also assessed migration and found that there was increased migratory property of these cells. We hypothesize that TGF‐β1 and BMP7 signaling may influence vascular thickening through modulation of expression of extracellular matrix proteins and cellular migrations. Our results suggest that the sexual differences could be attributable to an imbalance in TGF‐β1/BMP7 signaling, leading to venous fibrosis and venous neointimal hyperplasia in female AVFs compared with males (Figure S3).

There are limitations to the present study. First, the role of estrogen and its receptors needs to be investigated in future studies. Second, the mouse model may not simulate the clinical scenario. Finally, future studies in larger animal models need to be performed to investigate these findings.

In summary, in female AVFs, there is increased venous fibrosis mediated through an increase in TGF‐β1 and decrease in BMP7 pathway. This is accompanied by increased expression of smooth muscle cells and fibroblasts with venous neointimal hyperplasia and stenosis formation with decreased peak velocity. In cell culture, under hypoxic condition, female VSMCs have increased gene expression of TGF‐β1, TGF‐βR1, and Col1a with increased migratory capacity. In aggregate, these data suggest a role for TGF‐β1/BMP7 modulation in reducing venous stenosis formation in AVFs placed in females.

## Sources of Funding

This work was supported by National Institutes of Health HL098967 and DK107870 (Dr Misra).

## Disclosures

None.

## Supporting information


**Tables S1–S2**
**Figures S1–S3**
