# Supplementary material for: Differences in Transforming Growth Factor‐β1/BMP7 Signaling and Venous Fibrosis Contribute to Female Sex Differences in Arteriovenous Fistulas
Source: J Am Heart Assoc. 2020 Aug 6;9(16):e017420. doi: 10.1161/JAHA.120.017420 (PMC7660821; doi:10.1161/JAHA.120.017420)

SUPPLEMENTAL MATERIAL

Table S1. qRT-PCR primer design for genes.

Gene	Forward	Reverse
Tgfβ1	CGAAGCGGACTACTATGCTAAA	TCCCGAATGTCTGACGTATTG
TgfβR1	TTGCTCCAAACCACAGAGTAG	ACACTAAGCCCATTGCATAGAT
Bmp7	CAAGCAGCGCAGCCAGAATCG	CAATGATCCAGTCCTGCCAGCCAA
IL17Rb	AGCCGACTATTCAGTGTGGC	GTCTTGACGAGTTCCACTTGG
Col1a	GGTGAGCCTGGTCAAACGG	ACTGTGTCCTTTCACGCCTTT
Tubulin	ACCAACCTGGTACCCTACCC	AGGCATTGGTGATCTCTGCT
TBP1	AAGGGAGAATCATGGACCAG	CCGTAAGGCATCATTGGACT

Table S2. Antibodies used in this study.

Antibodies	Host	Catalog number	Provider	Dilution
IgG	rabbit	sc-2027	Santa Cruz	
α-SMA	mouse	ab7817	Abcam	1:400
α-SMA	rabbit	ab5694	Abcam	1:1000
Collagen I	rabbit	600-401-103	Rockland	1:5000
MYH11	rabbit	ab53219	Abcam	1:800
MMP-9	rabbit	ab38898	Abcam	1:1500
CD68	rabbit	ab125212	Abcam	1:2000
iNOS	rabbit	NB300-605	Novus Biologicals	1:2000
Arg-1	rabbit	NBP1-32731	Novus Biologicals	1:1500
CD31	rabbit	ab28364	Abcam	1:400
FSP-1	rabbit	07-2274	EMD Millipore	1:1000
FSP-1	Mouse	188-11191	Ray Biotech	1:500
HIF-1α	rabbit	ab2185	Abcam	1:1000
Ki-67	rabbit	ab9260	EMD Millipore	1:350
pSMAD3	rabbit	ab52903	Abcam	1:200
Alexa Fluor® 488	goat	ab150169	Abcam	1:1000
Alexa Fluor® 594	donkey	711-585-152	Jackson ImmunoResearch	1:1000
Alexa Fluor® 647	goat	A32728	Invitrogen	1:1000

Figure S1. Outline of the *in vivo* AVF study.

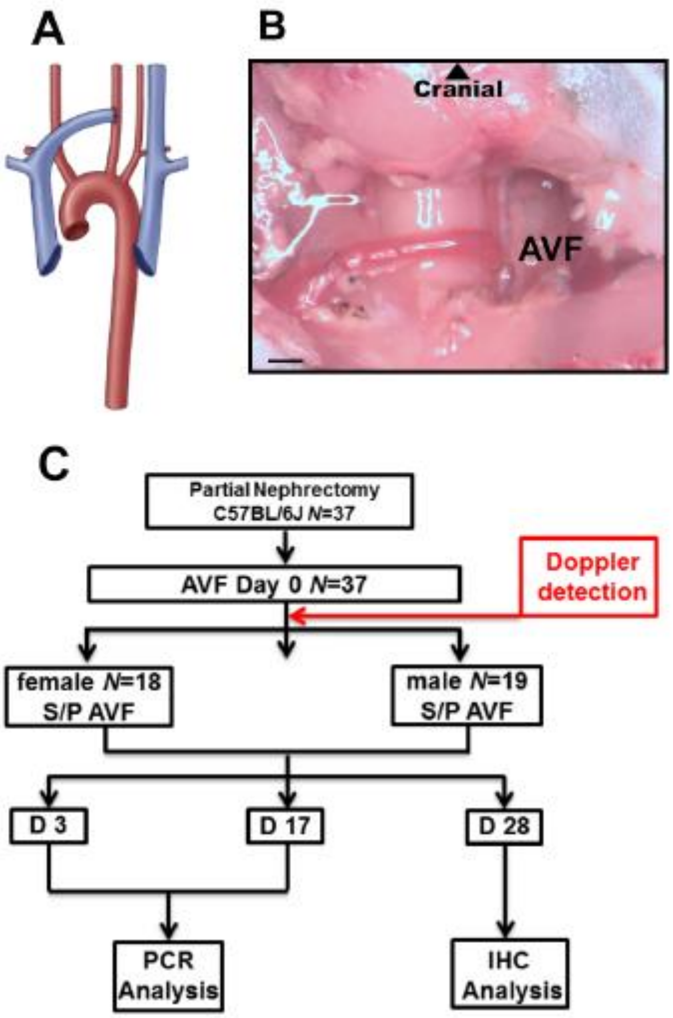

A total of 37 mice were utilized in the present study. (A) Schematic of autologous end-jugular vein to side-carotid artery AVF with anastomosis. (B) Intraoperative image of AVF. (C) Schema of female and male subgroups following partial nephrectomy and AVF surgeries. Scale bar is 1mm.

Figure S2. There was no significant sexual difference in HIF-1α staining.

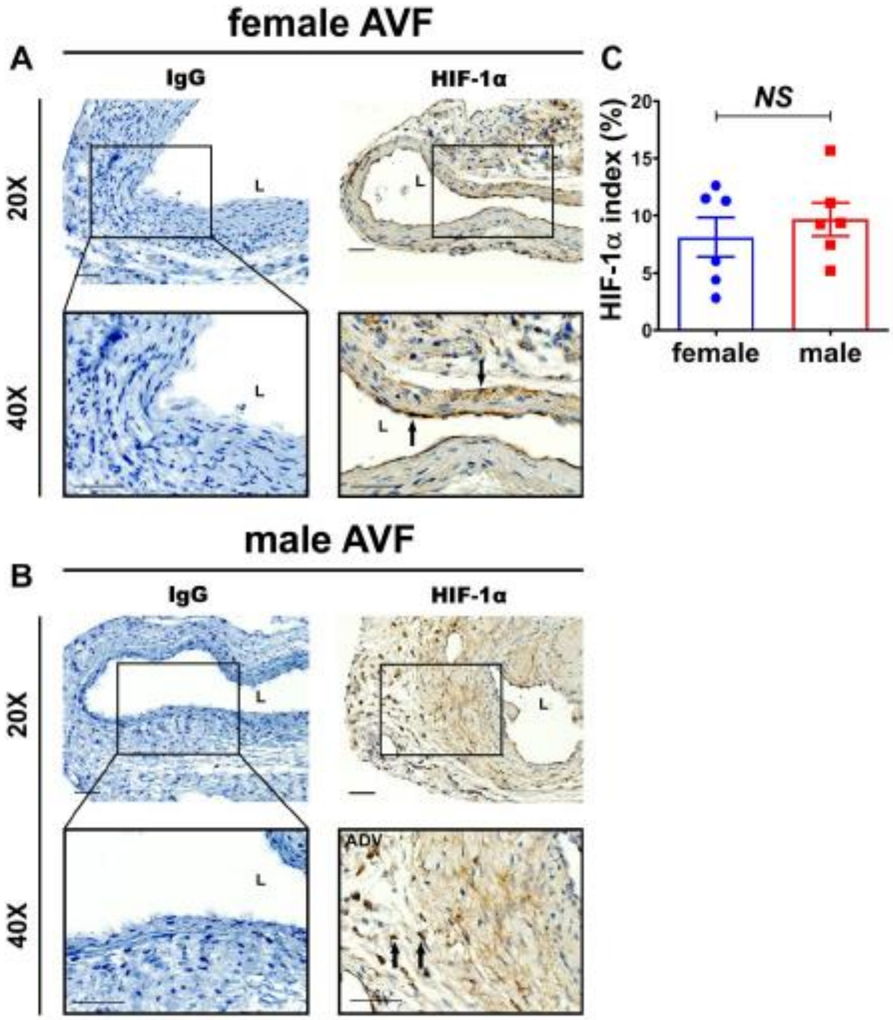

(A-B) Representative slides for negative IgG and positive HIF-1α staining in female and male outflow veins (female N=6, male N=6). Both female and male vessels presented positive HIF-1α staining in the whole vessel wall. (C) Semi-quantitative analysis showed no sexual difference in the average HIF-1α ($p>0.05$) index between female outflow veins and males. Two sample *t* test was performed. Significant differences are indicated *NS* $p>0.05$. Positive HIF-1α is stained as brown color. ADV, adventitia; L, lumen; solid arrows, positive cells. Scale bar is 50μm.

Figure S3. Conclusion of sexual differences in female and male AVFs.

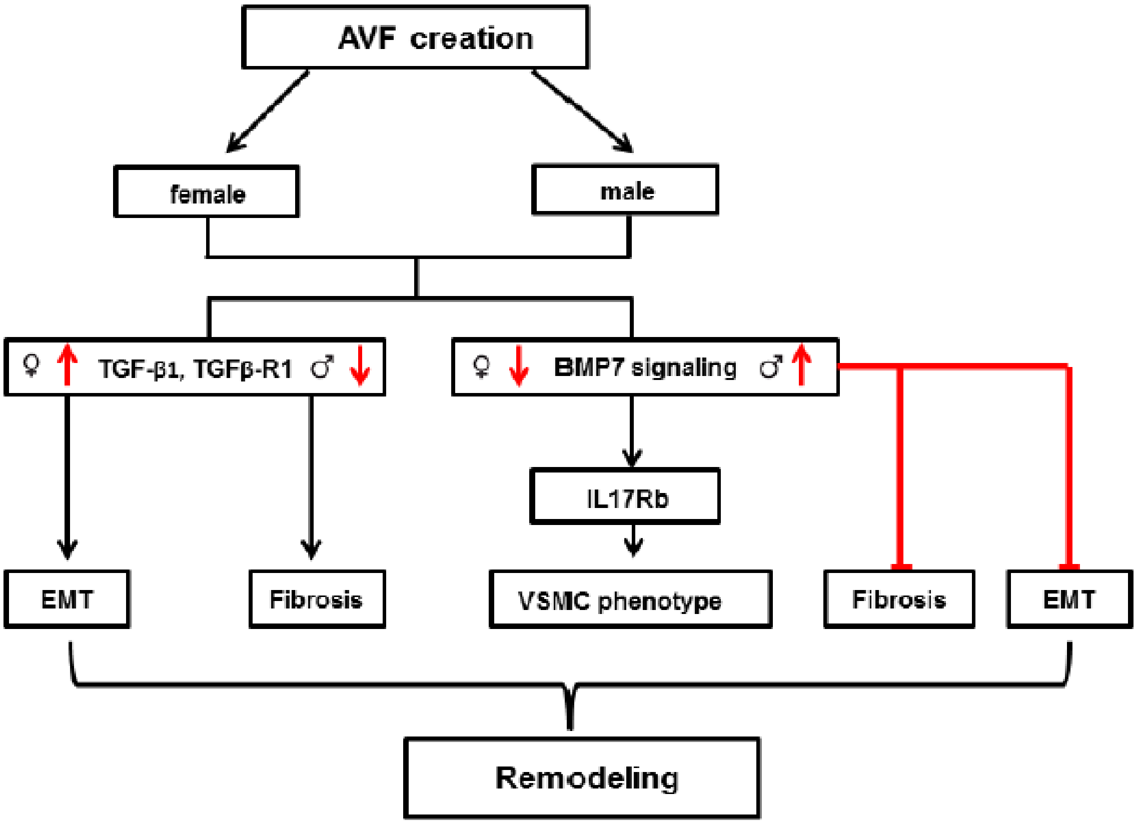

Supplement: Supplementary file 1 — Tables S1–S2 Figures S1–S3 [file JAH3-9-e017420-s001.pdf]
